# Early-career researchers in Chile: findings from a national survey on conditions, challenges, and institutional perceptions

**DOI:** 10.3389/frma.2026.1796649

**Published:** 2026-05-13

**Authors:** Alexia Nunez-Parra, Andrés E. Marcoleta, Cristóbal Feller, Rodrigo Herrera-Camus, Valentina Parra

**Affiliations:** 1 Laboratory of Cell Physiology and Neurobiology, Biology Department, Faculty of Science, Universidad de Chile, Santiago, Chile; 2 Frontiers Science Program 2022–2024 Cohort, Chilean Academy of Science, Santiago, Chile; 3 Grupo de Microbiología Integrativa, Biology Department, Faculty of Science, Universidad de Chile, Santiago, Chile; 4 Centro de Estudios Sociales Katalejo, Santiago, Chile; 5 Department of Astronomy, Universidad de Concepción, Concepción, Chile; 6 Millennium Nucleus for Galaxias (MINGAL), Concepción, Chile; 7 Laboratory of Cell Differentiation and Metabolism, Department of Biochemistry and Molecular Biology, Facultad de Ciencias Químicas y Farmacéuticas, Universidad de Chile, Santiago, Chile; 8 Advanced Center for Chronic Diseases (ACCDiS), Universidad de Chile y Pontificia Universidad Católica de Chile, Santiago, Chile; 9 SYSTEMIX Center for Systems Biology, Universidad de O'Higgins, Rancagua, Chile

**Keywords:** gender equity, public policies in science, research funding, science work conditions, work-family balance

## Abstract

Chile's investment in postgraduate training has produced a highly qualified cohort of early-career researchers. Yet little is known about how these researchers experience employment conditions, access to competitive funding, and the institutional environment in which they pursue their careers. Here, we report findings from a national survey of 267 early-career researchers working in Chile, examining training trajectories, employment conditions, grant-seeking experiences, time allocation, caregiving responsibilities, and perceptions of recent institutional reforms. Respondents reported high levels of intrinsic job satisfaction, particularly regarding intellectual challenge, autonomy, and the social value of their work. At the same time, they expressed concerns about contractual instability, workload intensity, and insufficient income. Nearly half held a second job, and those with caregiving responsibilities allocated less time to research. Care responsibilities were more frequently reported by women, who devoted substantially more time to caregiving than men. Participation in competitive funding programs varied across career stages, with higher application and success rates in entry-level schemes than in more advanced grant competitions. Gender differences were also observed in progression toward senior funding opportunities. Notably, a substantial proportion of unfunded proposals had received positive evaluations, suggesting that part of the country's research capacity remains unsupported despite being competitively viable. This reveals an unrealized scientific potential and a gap between Chile's expanding scientific base and the public investment available to sustain it. Although recent institutional reforms were perceived as contributing to greater diversity and regional participation, respondents identified limited improvements in overall funding levels and the international impact of Chilean research. These findings provide an empirical characterization of the working conditions and career challenges faced by early-career researchers in Chile, highlighting persistent structural barriers related to career consolidation, funding access, gender equity, and work-life balance.

## Introduction

In recent decades, the global academic landscape has undergone profound transformations driven by neoliberal policies, intensified competition, and persistent funding constraints. Young researchers, especially those transitioning from postdoctoral roles into early faculty positions or equivalents, increasingly encounter a work environment characterized by instability, excessive workloads, and mounting pressure to perform. Numerous studies report that researchers and early-career researchers in particular experience short-term contracts, limited institutional support, and overwhelming expectations to maintain high publication rates while simultaneously balancing teaching, research, and administrative responsibilities. Productivity metrics and the competition for funding and permanent positions dominate nearly every aspect of academic life (Allen et al., 2024; Pietilä, 2019).

A recurring theme across disciplines is the prevalence of precarious employment arrangements. Whether in STEM fields, the social sciences, or the humanities, early-career researchers are frequently hired on temporary or fixed-term contracts that offer no clear path to tenure. Performance is often measured by publication counts and grant acquisition (Christian et al., 2021; Holley et al., 2018), and the uncertainty of national funding cycles, combined with reliance on project-based grants, exacerbates stress and undermines personal well-being. Excessive workloads compound these pressures: newly appointed faculty commonly balance large teaching loads with managing research groups, writing grant proposals, and meeting administrative demands, leaving little time for research during the critical early years of their careers (Larson et al., 2019). In Latin America, these challenges are often compounded by more limited research funding, higher levels of labor informality, and uneven institutional capacity across countries, although national contexts vary in terms of investment and system maturity.

Chile's scientific landscape mirrors these global trends while adding its own dynamics: it exhibits budgetary constraints, uneven resource allocation across Chilean regions, and structural as well as institutional limitations. Chile, an OECD (Organization for Economic Co-operation and Development) member, holds the second-to-last place in Science and Technology expenditure, investing 0.39% of GDP, which is much lower than the OECD average of 2.9% (World Bank, 2025). Despite this, Chile maintains a relatively stable and institutionalized research funding system within the Latin American context, supported by national agencies and competitive grant programs.

In recent years, Chile has attempted to recalibrate its scientific priorities with the establishment of entities such as the Ministry of Science, Technology, Knowledge and Innovation (Ley N° 21.105, 2018). Despite these institutional reforms, research funding remains insufficient to keep pace with the rapid growth of research outputs. Although new grants aimed at applied, collaborative, and transdisciplinary research have been introduced in line with the Ministry's strategic vision, these initiatives have not come with an overall budget increase; rather, funds have been reallocated from basic-research programs (most notably the longstanding FONDECYT program) toward newer calls (Agencia Nacional de Investigación y Desarrollo, 2025).

Opinions on this shift are divided. Some welcome the move toward socially engaged, mission-oriented science; others worry about its long-term implications for researchers whose work depends on stable support for fundamental inquiry or basic science. Importantly, Chile's policy changes are part of a broader global transformation: early-career researchers worldwide must now navigate increasingly complex funding models, shifting collaboration frameworks, and productivity expectations for which they were not originally trained, adding further pressure to an already precarious academic environment.

In this study, early-career researchers are defined as individuals holding a PhD who have initiated their academic or research careers within the past 10 years. This operational definition is consistent with eligibility criteria used in major national funding programs (e.g., early-career grant schemes), while also encompassing a heterogeneous group in terms of career progression and institutional position. In the Chilean context, early-career researchers face additional structural challenges. Over the past decade, the number of Chilean PhD holders and early-career researchers launching independent lines of research has increased substantially, fueled by the expansion of national doctoral programs and by the Becas Chile fellowship scheme, which has financed thousands of doctoral and postdoctoral trajectories abroad (Nunez-Parra and Ramos, 2014). However, the program's requirement for recipients to return and work in Chile for a specified period has placed further strain on a system already characterized by limited institutional capacity and scarce research funding. As a result, competition for stable positions and project-based resources has intensified, heightening precarity and constraining the country's scientific development.

While international reports offer valuable perspectives on systemic barriers such as funding, mobility, and professional development, country-specific data remain scarce, particularly in systems that differ substantially in levels of investment and institutional capacity. To address this gap, and using international evidence as contextual reference rather than a basis for direct comparability, we surveyed 267 early-career researchers, gathering insights into their professional experiences and perceptions of the national scientific system. The survey results reveal that Chile's postgraduate funding has produced a well-qualified and motivated cohort of scientists who value the intellectual challenge and social purpose of their work. However, persistent downstream problems, including contractual precarity, limited program capacity, and caregiving-related time constraints, limit career consolidation, reduce competitiveness for funding, and exacerbate inequalities. These findings suggest the potential value of targeted actions, including expanded and bridge funding, formalized postdoctoral contracts, and protected research time for caregivers, to help translate training investments into sustained and more equitable research capacity.

Together, these data allow for an empirical characterization of early-career researchers' working conditions in Chile and provide insight into how current institutional arrangements shape career trajectories. The findings also point to emerging tensions between the expansion of postgraduate training and the availability of stable career opportunities and research funding, raising questions about the capacity of the system to fully support its growing scientific workforce.

## Methodology

The study involved administering a survey to 267 early-career researchers in Chile. The target population was operationally defined as individuals meeting the following criteria: (i) holding a doctoral degree; and (ii) having begun work as an academic or researcher at a university, research center, foundation, or company in Chile up to 10 years prior to the survey administration (regardless of whether they were employed at such institutions at the time of responding to the survey).

The questionnaire was designed by the research team based on the study's objectives and covered five dimensions: (i) sociodemographic characteristics of respondents, (ii) academic or professional trajectory, (iii) access to competitive funding, (iv) perceptions of Chile's scientific institutional framework, and (v) job and personal satisfaction. For the latter dimension, a question from the Early Career Researcher Survey conducted in 2022 by the Swiss National Science Foundation was adapted (Swiss National Science Foundation, 2022).

The survey was administered between October 23 and December 23, 2024, via a self-administered online form using the open-source tool LimeSurvey. During this period, the study was promoted, and participation was encouraged among the academic community and the general public using a non-probabilistic snowball sampling approach, through universities, scientific societies, media outlets, and social media. The configuration of the online survey platform was designed to prevent progression unless mandatory questions were answered, thereby minimizing the number of missing responses. In instances where missing data were present, these were explicitly coded as “No Response” and were not imputed to any alternative category.

As a non-probabilistic sample, the respondents are not intended to be statistically representative of the entire population of early-career researchers in Chile. Participation was voluntary and may be influenced by self-selection biases, including higher engagement from individuals with stronger opinions about the research system. In addition, the distribution of respondents across disciplines and geographic regions reflects the dissemination channels used and may not fully capture the diversity of the national research community. Therefore, the findings should be interpreted as reflecting the experiences and perceptions of the surveyed group rather than as population-level estimates.

The final sample consisted of 267 responses. Data analysis was conducted using SPSS software, employing univariate and bivariate analyses, including Chi-squared tests and Mann-Whitney *U* tests. Exact *p*-values are reported, given the exploratory and descriptive nature of this research, and no formal correction for multiple testing was applied.

## Results

### Sample and study overview

**Figure 1:**
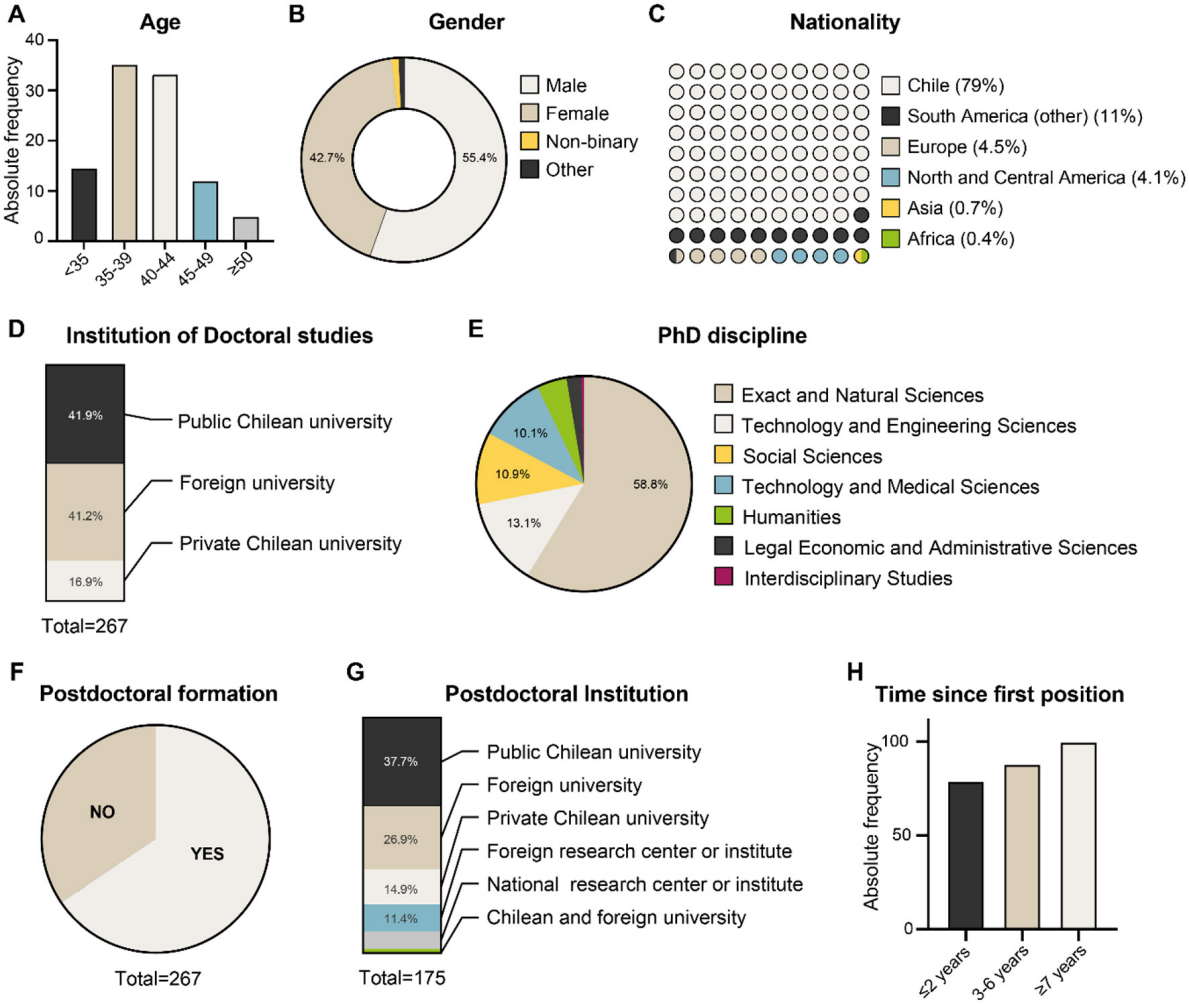
Demographic and academic formation of the respondents included in the present study. **(A)** Age, **(B)** gender, **(C)** nationality, **(D)** institution of doctoral studies, **(E)** PhD discipline, **(F)** postdoctoral formation, **(G)** postdoctoral institution, and **(H)** time since first position.

The survey was answered by 267 early-career researchers currently working in Chile. 55.4% of the respondents declared themselves to be male, 42.7% female, and 1.8% non-binary or “others” (Figure 1). Also, most of the individuals (68.5%) were in the range of 35–44 years old, with 14.6% being 35 years old or less, and 16.9% 45 years old or more. Seventy-nine percent were born in Chile (of whom 3.4% belong to an original Chilean ethnicity), 11.2% were from another South American country, 4.5% from Europe, 4.1% from North or Central America, and 1.1% from Asia or Africa. Regarding the research area they specialized in, most of the respondents reported holding a doctorate degree from the Natural Sciences area (58.8%), followed by Technology and Engineering (13.1%) and Social Sciences (10.9%). The remaining participants hold a PhD in Technology and Medical Sciences, Humanities, Legal, Economic, and Administrative Sciences, and Interdisciplinary studies. Overall, the sample reflects a heterogeneous but predominantly STEM-oriented early-career researchers concentrated in the metropolitan region.

### Academic trajectory and working conditions

Concerning the trajectory after finishing the PhD, a total of 250 (93.6%) respondents reported receiving fellowships to support their postgraduate studies. Among them, 70.8% were funded by scholarships awarded by the National Agency of Research and Development (ANID). Nearly 60% obtained their doctoral degrees from Chilean universities, while 40% completed postdoctoral training abroad at universities or research centers. There is a high correlation (Chi-squared test; *p* ≤ 0.005) between the country where the PhD was obtained and the country where the survey participants did their postdoctoral studies (Of those who obtained their doctoral degree from a Chilean university, 72% pursued a postdoctoral position in Chile, whereas 62% of those who earned their doctorate abroad also completed their postdoctoral training abroad, in institutions outside of Chile; Figure 1).

**Figure 2:**
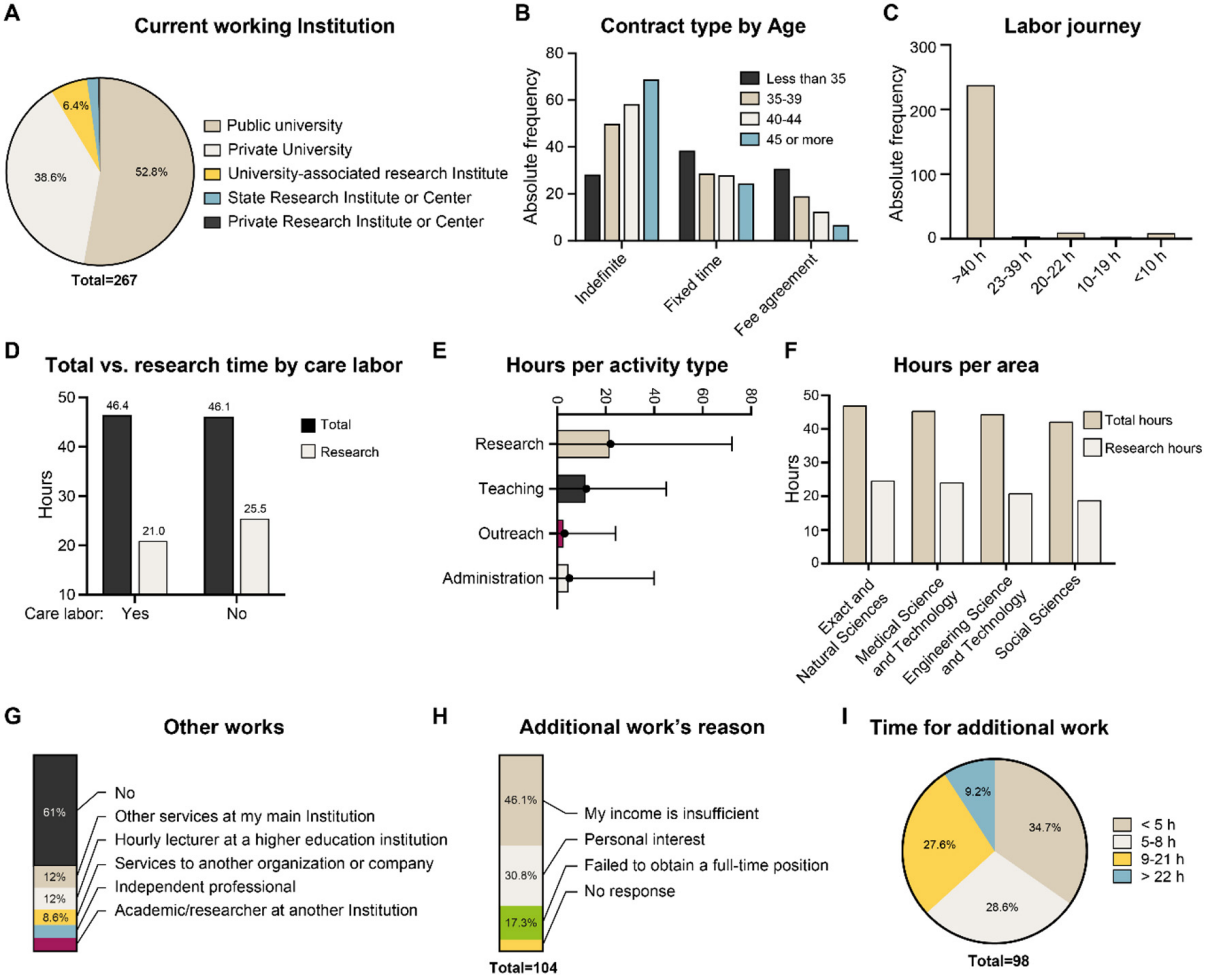
Working conditions of early-career researchers in Chile. **(A)** Current working institution, **(B)** Contract type by age, **(C)** labor journey, **(D)** total vs. research time by care labor, **(E)** hours per activity type, **(F)** hours per area, **(G)** other works, **(H)** additional work's reason, and **(I)** time for additional work.

All participants are currently employed at academic or research institutions. Public universities account for the largest share, employing approximately 53% of respondents, while 38.6% work at private universities (Figure 2). Our study found that 89.5% of researchers hold full-time positions (defined as 40 h per week or more), and 95.5% are employed either full- or part-time (22 h per week or more). However, only 52.8% have indefinite contracts, and 16.5% work under the “*honorario*” regime, which involves fee-based employment. Notably, the likelihood of having an indefinite contract increases with the researcher's age (Chi-squared test; *p* = 0.049). As in other professional sectors, the Metropolitan Region hosts most researchers, with 61% of survey participants working there. The Valparaíso Region follows, with 13.1% of respondents.

Interestingly, 49% of researchers reported holding a second job. When asked about their motivations, most cited insufficient income from their primary role as researchers or professors (Figure 2). This result applies not only for researchers who are hired part-time, but for 34% of respondents who are hired full-time. In this case, they devote a median of 5 h per week to this additional job. The types of secondary employment vary across individuals: providing additional services at the university or research center that are not included in their contract; working as an hourly lecturer at another higher education institution; providing services to another organization or company, not as a researcher; working independently; and/or working as an academic or researcher at another university or research center. It is worth noting that there is not a predominant secondary job alternative (all of them were mentioned by 7 and 12% of respondents) and that 30.8% of people having a second job indicated that personal interest was a driving factor behind their additional employment.

Regarding weekly time allocation to academic activities there was a great variation among respondents, who spend a median of 22 h on research, 12 h on teaching, 3 h on science communication, and 5 h on administrative duties (e.g., career coordination, departmental council participation). Although male and female researchers work on average nearly identical weekly hours (46.4 vs. 46.1 h) and dedicate similar time to research (23.3 vs. 23.7 h/week), those with caregiving responsibilities spend, on average, four fewer hours per week on research (Mann-Whitney *U* test; *p* = 0.02).

Finally, although not statistically significant, there is a trend indicating that researchers employed at public universities report the longest workweeks, averaging 47.3 h per week, compared to 45.3 h at private universities and 43.6 h at research institutions.

Taken together, these results indicate that high levels of formal employment coexist with significant contractual instability, income fragmentation, and uneven time allocation, pointing to structurally constrained early-career trajectories.

### Access to funding for scientific research

**Figure 3:**
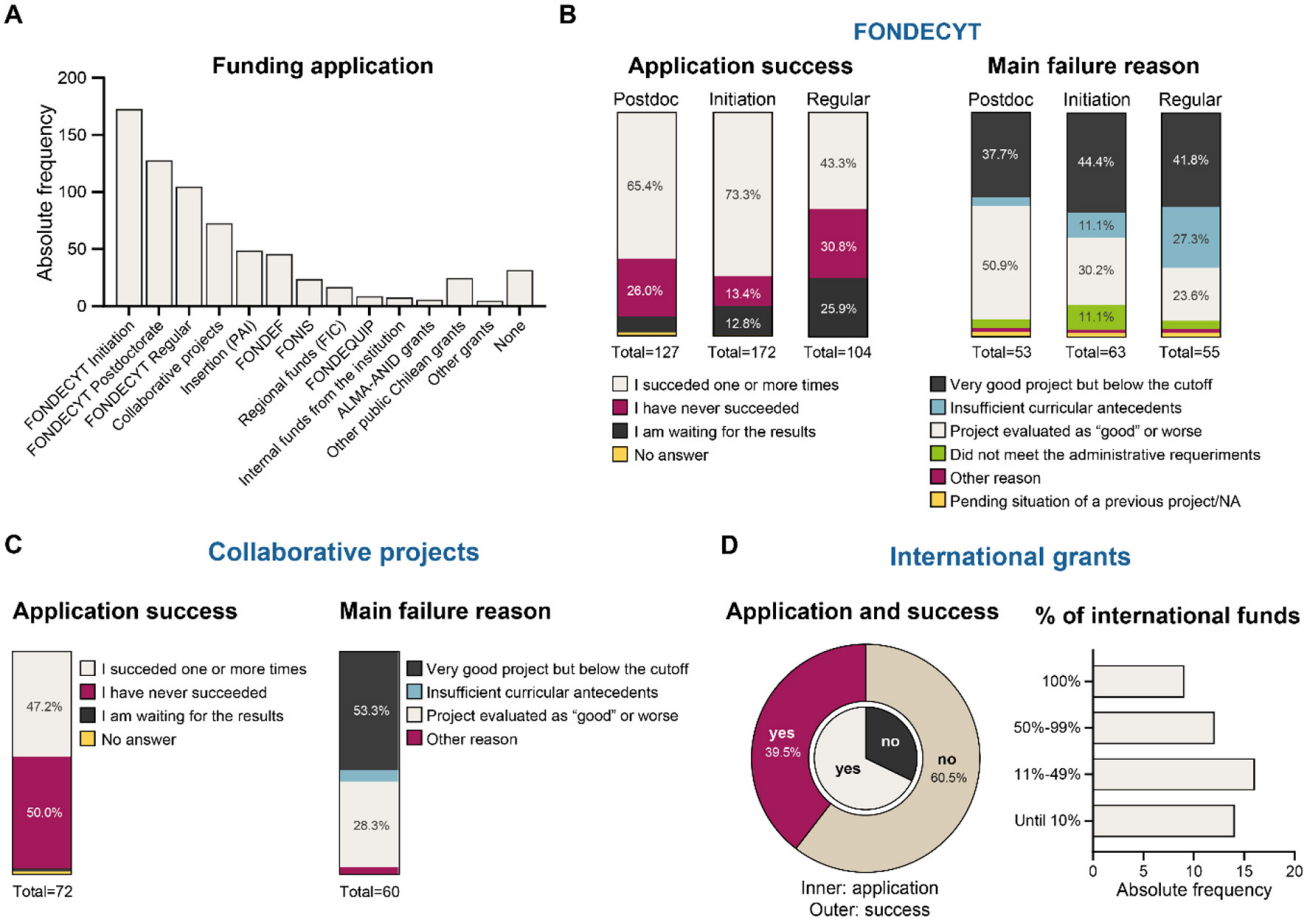
Funding application and success in early-career researchers working in Chile. **(A)** Application to main Chilean grants, **(B)** FONDECYT grants, **(C)** collaborative projects, and **(D)** international grants.

We explored the experience of the respondents in applying to and getting funding for their research through the main Chilean scientific grant programs managed by ANID (https://anid.cl/). These programs included: (a) the three grants from the National Fund for Scientific and Technological Development (FONDECYT) program, directed to either postdoctoral researcher (FONDECYT *Postdoctoral*), early career scientists (FONDECYT *Initiation*, only for those researchers that have finished their PhD in the last 10 years), or mid-career plus senior researchers (FONDECYT *Regular*); (b) collaborative research grants (e.g., “Millennium Institute” and “Millennium Nucleus” grants, “Ring” grants); (c) applied science grants (e.g., FONDEF, FONIS, etc.); and (d) international grants (Figure 3).

Regarding FONDECYT participation, 64.4% of respondents reported having applied to the *Initiation into Research* grant, 47.6% to the *Postdoctoral* grant, and 39% to the *Regular* grant. These figures highlight the substantial barrier to progressing beyond the Initiation stage and achieving competitiveness in the Regular Program. Applications to the *Initiation* grant correlated positively with having completed a postdoctoral fellowship (72.6 vs. 48.9%; Chi-squared test; *p* ≤ 0.005) and with greater seniority as an academic or researcher (72% among those with seven or more years of experience; Chi-squared test; *p* = 0.008). A considerable majority (73.3%) declared success in obtaining the *Initiation* grant (awarded only once per individual), whereas 13.4% reported one or more unsuccessful attempts, and 12.8% were still awaiting the results of the 2024 call at the time of this survey. Notably, among the 63 respondents who declared at least one failed application to the *Initiation* grant, 44.4% reported that, despite falling below the funding cutoff, their proposals had been evaluated as “very good” (scores between 4.0 and 5.0 on a 1–5 scale). According to FONDECYT's evaluation criteria, this range corresponds to projects that meet the assessed criterion in a very good to excellent manner, with only minor weaknesses. The full FONDECYT evaluation scale is as follows: 0–0.9 Not eligible (insufficient or incomplete information), 1.0–1.9 Deficient, 2.0–2.9 Fair, 3.0–3.9 Good, 4.0–4.9 Very good, and 5.0 Excellent. This distribution underscores that many scientifically solid proposals remain unfunded due to the limited budgetary capacity of the program. Together, these patterns reveal a progressive bottleneck in access to competitive funding, where advancement increasingly depends on seniority, prior funding success, and employment stability.

Among those who have never applied to the *Initiation* grant, the main reasons cited were an insufficient CV (29.5%) and difficulties meeting administrative requirements (22.1%), followed by 6.5% who opted to apply directly to the Regular grant.

Regarding the FONDECYT *Postdoctoral* grant, 65.3% succeeded in obtaining the funds, 26% failed one or more times, and 7.1% were still waiting for the results of the 2024 call at the time of this survey. In this case, although most of the projects were rejected for having a mark below 4.0 (50.9%), still, 37.7% of the projects were evaluated as “very good.” A substantial proportion of the people who have never applied for the *Postdoctoral* grant performed their postdoc in a foreign country (30.7%), 22.1% indicated “other reasons,” while 15% were “not interested.” The *Postdoctoral* grant application correlated negatively with seniority, where 50.6% of the applicants had 2 years or less of experience as an academic or researcher, 36% of those who had 7 or more years of academic experience had applied to this grant. Also, 65.9% of the applicants had a “Fee agreement” with the respective institution, which does not involve a formal labor contract and thus does not provide labor benefits. This issue reflects the frequent precariousness of the postdoctoral researchers in Chile.

Concerning the FONDECYT *Regular* grant, 43.3% have succeeded one or more times, while a high proportion (30.8%) have failed all attempts, and 26% were waiting for the results of the 2024 call at the time of this survey. As in the other FONDECYT programs, 41.8% declared, as the main reason for rejection, that the project was evaluated as “very good” but fell below the adjudication cutoff. Moreover, 60.7% of the individuals who have never applied to the Regular grant declared, as the main reason, having an insufficient CV, reinforcing the strong barrier that this grant means to early- and mid-career scientists. Of note, the application to FONDECYT *Regular* grants significantly correlated with gender (48% male vs. 27.2% female; Chi-squared test; *p* = 0.001), age (40 years old or more; Chi-squared test; *p* ≤ 0.005), and having performed postdoctoral research (44.6 vs. 28.3%; Chi-squared test; *p* = 0.009). Regarding this last point, a strong positive correlation was observed between applying for the *Regular* grant and having performed the postdoctoral research in a foreign university (66%) or a foreign institute or research center (60%; Chi-squared test; *p* = 0.003). Also, the percentage of researchers applying to FONDECYT *Regular* projects increases with academic or research experience seniority: 11.4% of researchers with two or less years of experience have applied to this grant, while 59% of researchers having 7 or more years of experience have applied. Regarding the labor situation, the percentage of researchers varies with contract type: 50.4% of researchers with indefinite contract has applied, while 38.5% of researchers with a defined time period contract has applied and 6.8% of researchers under the “honorario” regime has applied.

Compared to FONDECYT, a more limited group (27%) has applied to collaborative research projects. From them, 50% have failed all their attempts, and 47.2% have succeeded one or more times. Also, in this kind of grant, the most common failure reason (53.3%) was that, despite the project being evaluated as “very good,” it fell below the adjudication cutoff. A high proportion of the individuals who have never applied to collaborative projects indicate an insufficient CV (35.9%) or a low success expectation (13.8%) as the main reasons. Collaborative project application significantly correlated with being 40 years old or older (Chi-squared test; *p* = 0.009), having a career as a researcher or academic of 7 years or more (Chi-squared test; *p* ≤ 0.005), and having an indefinite contract (Chi-squared test; *p* = 0.006), demonstrating that more experienced researchers target this kind of grant.

Similarly, international funds are not frequently targeted by early-career scientists working in Chile, as 67.8% of the respondents declared never having applied to this kind of grant. Nevertheless, most of those who applied one or more times (60.5%) succeeded in obtaining funding, some (17.6%) declaring that 100% of their research budget comes from international funds. These observations suggest that international funds may represent underexploited opportunities within the current funding landscape.

Overall, the funding landscape suggests that a substantial portion of competitively viable research projects remains unsupported, reflecting structural constraints that limit the translation of available scientific capacity into funded research.

### Perception of the National Research Agency and the Ministry of Science, Innovation, Knowledge and Technology

**Figure 4:**
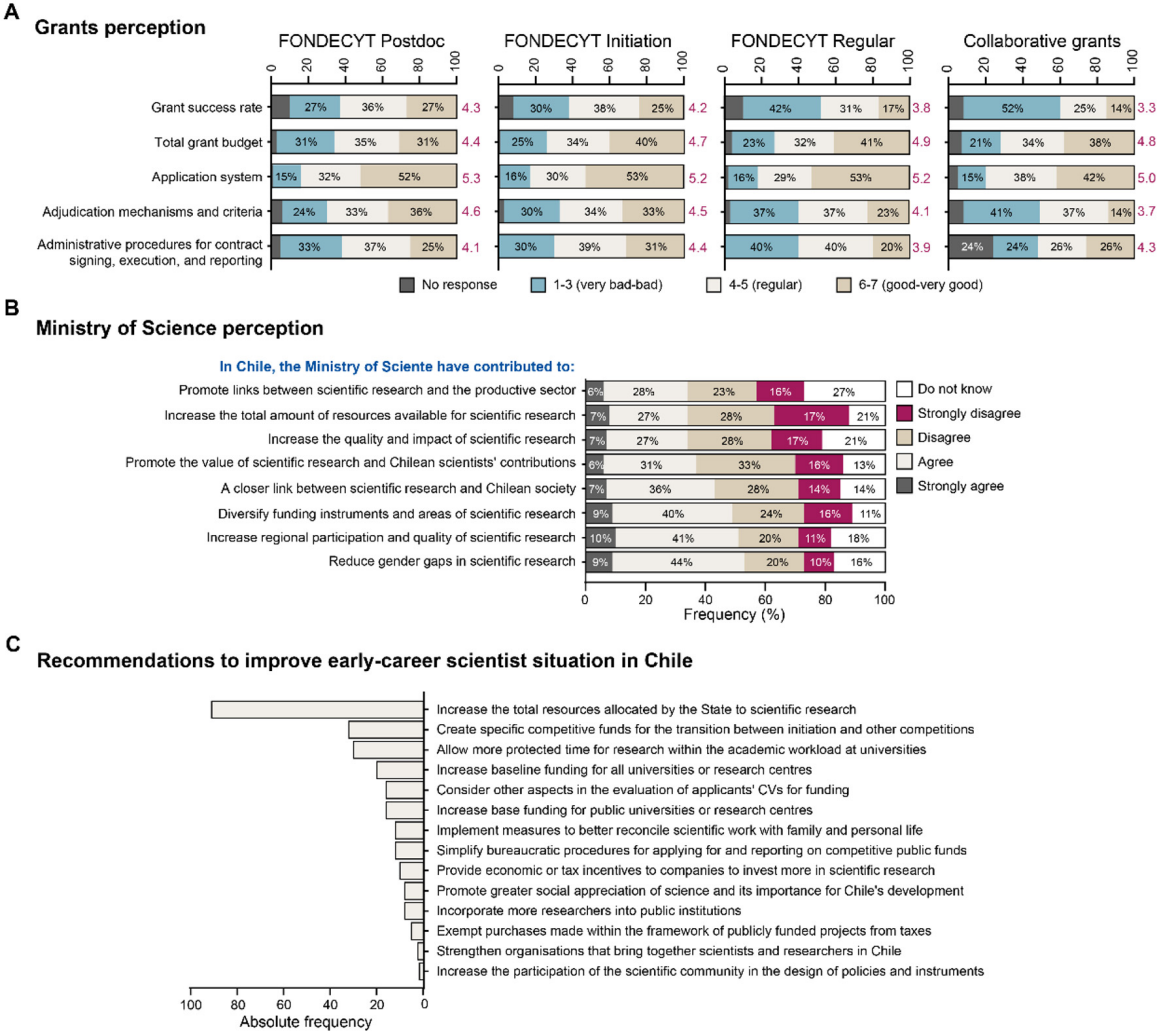
Perception of the Chilean Institutionality for science and technology. **(A)** Grants perception, **(B)** Ministry of Science perception, and **(C)** recommendations to improve early-career scientist situation in Chile.

We asked respondents to rate their experience applying to FONDECYT and collaborative programs on a 1–7 scale (7 = best), which is widely used in school and higher education in Chile. The application platform received the highest ratings, with all program application processes scoring above 5 (Figure 4). Perceptions of the percentage of projects funded showed the greatest variation across programs: FONDECYT *Initiation* and *Postdoctoral* programs received average scores of 4.2 and 4.3, respectively, while FONDECYT *Regular* and collaborative projects scored lower at 3.8 and 3.3, reflecting diminishing satisfaction with funding probability as researchers advance in their careers. Perceptions of the funding amounts awarded were consistent across programs, with an average score of 4.7.

Respondents evaluated the impact of the Ministry of Science, Innovation, Knowledge and Technology (established in 2018) using a five-level agreement scale; overall, more than half of the respondents indicated that the Ministry has contributed to decreasing the gender gap and to increasing participation and research quality outside the Metropolitan Region, 49% agreed that it has diversified scientific programs and research areas, and 43% agreed that it has promoted stronger ties between scientific research and society. In contrast, perceptions were notably more negative regarding resources and scientific performance, with 54% disagreeing or strongly disagreeing that the Ministry has increased funding for Chilean research and 45% disagreeing or strongly disagreeing that it has improved the quality and impact of Chilean science.

These results suggest a perceived mismatch between improvements in institutional structure and limited progress in funding capacity and research outcomes.

### Recommendations from early-career researchers

To collect the recommendations of early-career researchers to improve their situation, we asked the respondents to prioritize different aspects of the scientific system (Figure 4). 55.7% of them expressed that it is necessary to increase the public budget destined to science, followed by 33.6% who suggested that it would be beneficial to develop research programs or funding designed specifically for researchers who have already obtained FONDECYT *Initiation* and are transitioning into the FONDECYT *Regular*. These suggestions are followed by “having more protected time to dedicate to research activities among the academic duties” (28.7%), to “consider other criteria to evaluate the CV of the applicants besides number and quality of articles published” (24.5%), and “increasing the basal funding for universities and research institutions” (2.6%).

These priorities reflect a consistent emphasis among respondents on strengthening funding capacity, improving career progression pathways, and expanding structural support mechanisms.

### Care responsibilities and gender inequality

**Figure 5:**
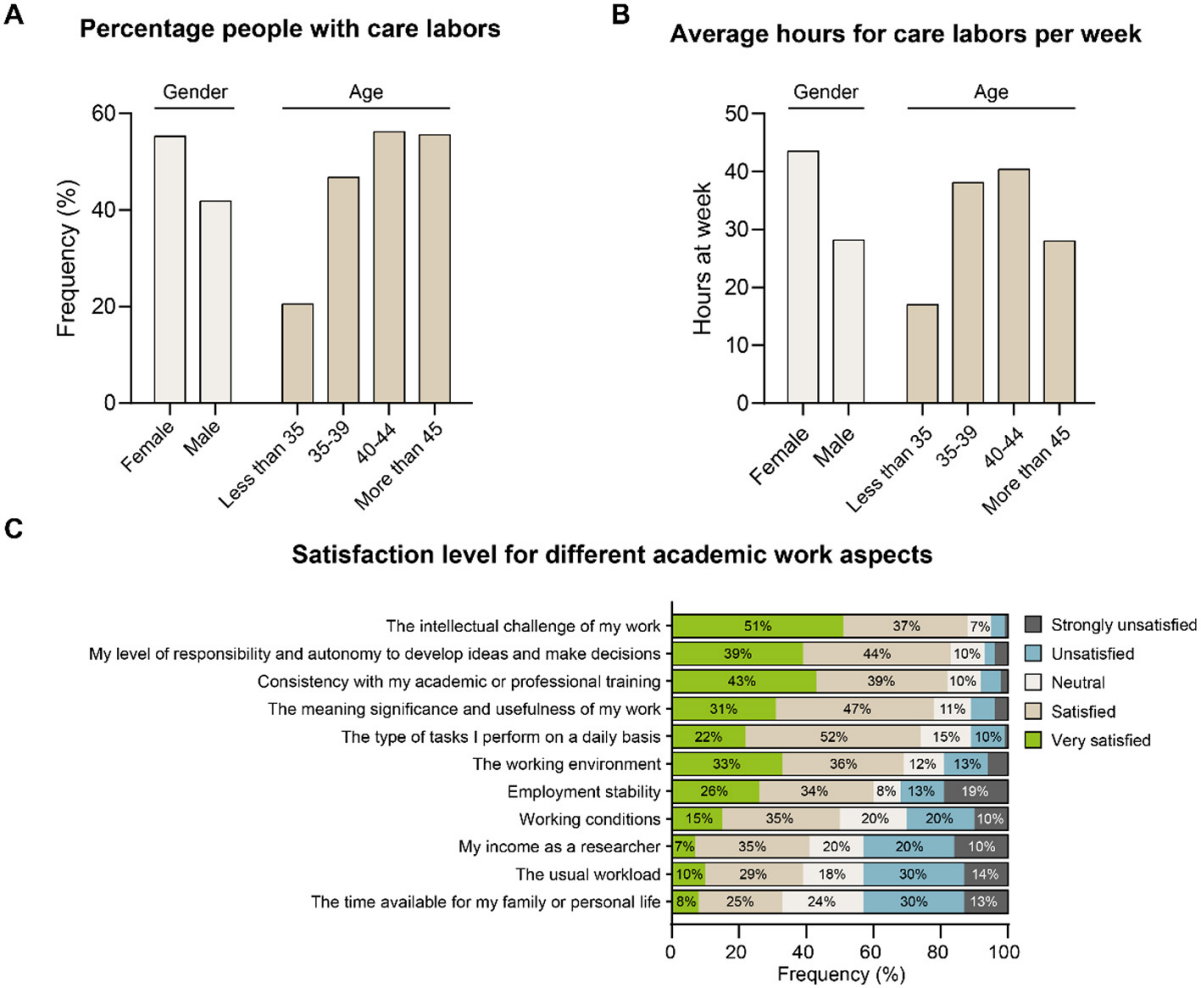
Care responsibilities, gender inequality, and satisfaction levels. **(A)** Percentage people with care labors, **(B)** average hours for care labors per week, and **(C)** satisfaction level for different academic work aspects.

Early-career researchers commonly have to make compatible career progression with care responsibilities (Figure 5). However, a significantly higher proportion of female scientists (46% of women vs. 28.2% of male respondent, Chi-squared test; *p* = 0.032) reported caring for someone, and there is a pronounced gender difference in care time, with women spending on average 43.5 h per week on childcare or adult care duties compared with 28.2 h for men (Mann-Whitney *U* test; *p* = 0.02). Despite this disparity, female and male scientists report similar total weekly working hours (45.3 vs. 47.4 h per week) and devote a comparable share of their working time to research activities (23.3 vs. 23.7%). The most notable gender gap in career advancement observed in the survey concerned applications to the FONDECYT *Regular* program, as we previously mentioned, with only 27.2% of female respondents having applied at least once compared with 48.0% of male respondents (Chi-squared test; *p* = 0.001).

These findings suggest that caregiving responsibilities introduce systematic time constraints that may contribute to cumulative disadvantages over time in accessing advanced funding opportunities.

### Work and personal fulfillment

To assess work fulfillment, participants were asked to rate their satisfaction across various aspects of their professional lives. A notable 88% reported being satisfied or very satisfied with the intellectual challenge their work entails (Figure 5). They also expressed high appreciation for the level of responsibility, autonomy in developing projects and making decisions, and the sense of purpose and social impact associated with their roles. However, researchers were less satisfied with their available time for family life, overall workload, and salary. Interestingly, responses showed a consistent pattern across all categories analyzed, regardless of gender, contract type, or institutional affiliation.

This pattern indicates that high levels of intrinsic motivation coexist with structural dissatisfaction related to workload, compensation, and work-life balance.

## Discussion and conclusion

The results indicate that while Chile's postgraduate training system successfully funds and produces a large cohort of researchers, structural vulnerabilities emerge during career consolidation, and precarious contract forms persist despite high rates of full- and part-time employment, a scenario that mirrors patterns documented in the Latin American region (Miranda-Nieto et al., 2022). Nearly half of respondents held a second job, often to supplement insufficient primary income; even among full-time staff, a median of 5 h per week was diverted from core research duties, fragmenting effort across activities. Comparable patterns have also been observed in the Early Career Researcher Survey conducted by the Swiss National Science Foundation, where over 40% of respondents reported employment instability or reliance on multiple affiliations, and nearly one-third indicated that excessive administrative burdens detracted from research time (Swiss National Science Foundation, 2022). In both contexts, insufficient remuneration and short-term contracting remain key barriers to research continuity, with measurable negative implications for work-life balance and academic productivity. In the Chilean case, caregiving responsibilities are associated with reduced research time: caregivers allocate approximately four fewer research hours per week despite similar total working hours across genders. This situation resonates with the Swiss findings, where women were significantly more likely to report time pressure linked to family obligations and unequal access to flexible work arrangements (Swiss National Science Foundation, 2022). Together, these findings point to a structural mismatch between strong upstream investment in researcher training and downstream conditions, characterized by contractual insecurity, income insufficiency, geographic concentration, and care-related time constraints, that limit career progression and exacerbate gendered inequities. Also, they underscore the need for policies that ensure contract stability, institutional childcare support, and workload alignment with remunerations, in line with the *Agenda de Mejor Trabajo en Investigación's* emphasis (Agenda for Better Research Work) on equitable and sustainable career pathways (Ministerio de Ciencia, Tecnología, Conocimiento e Innovación, 2024).

### Early-career researchers and funding landscape

The funding landscape revealed by respondents underscores a strong dependence on ANID-managed programs but also reveals a pronounced funnel that privileges seniority, international mobility, and secure employment while leaving many high-quality proposals unfunded. High application and success rates at the Initiation level contrast sharply with lower uptake and more stringent barriers for Postdoctoral and Regular competitions, where applicants are disproportionately male, older, internationally trained, and formally contracted. This “bottleneck effect,” where advancement increasingly depends on seniority, mobility, and prior funding, echoes the pattern documented in Switzerland, where early-career researchers expressed high satisfaction with the intellectual challenge and autonomy of their work, yet only 37% felt confident about securing long-term research positions (Swiss National Science Foundation, 2022). In both systems, limited funding and structural precarious conditions create discontinuous trajectories that hinder the consolidation of independent research programs. It is important to mention that direct comparisons between the Chilean and Swiss contexts must be interpreted with caution. Switzerland invests approximately 3.1% of GDP in R&D compared to Chile's 0.39%, and Swiss early-career researchers benefit from a well-institutionalized funding framework and stronger federal labor protections. In Chile, funding is concentrated primarily in FONDECYT, labor precarity (including widespread use of the “honorario” regime) is structurally embedded, and institutional capacity can vary significantly across regions. Despite these profound socio-economic differences, our decision to use data from the Swiss National Science Foundation (SNSF), a survey whose geographical coverage encompasses all researchers affiliated with Swiss institutions, regardless of their nationality, was driven strictly by methodological compatibility. The SNSF study employed a survey structure highly similar to ours, allowing for a direct, item-by-item contrast of early-career conditions. To our knowledge, equivalent studies utilizing this specific methodological framework are currently unavailable for other Latin American countries.

We acknowledge that benchmarking against an OECD nation and a robust scientific power like Switzerland inherently highlights Chile's systemic gaps rather than its regional strengths. Viewed from a Latin American perspective, Chile actually holds a privileged position; due to its economic performance, the country faces comparatively fewer critical funding deficits than many of its neighbors, maintaining a stable framework of state funding mechanisms through the National Agency for Research and Development (ANID; United Nations Educational, Scientific and Cultural Organization, 2021; Red de Indicadores de Ciencia y Tecnología Iberoamericana e Interamericana, 2023). Nonetheless, despite Chile's regional stability and the stark macro-economic contrast with Switzerland, the thematic parallels across both systems suggest that early-career researcher precarity is a structural phenomenon that transcends specific national contexts and funding capacities.

The Chilean data further suggest that even highly rated proposals frequently remain unfunded due to budgetary constraints, indicating that a portion of competitively viable research capacity remains unsupported. This finding is consistent with ANID's own recognition of underfinancing and the need for bridge mechanisms and intermediate funding stages in the *Agenda de Mejor Trabajo en Investigación* (Ministerio de Ciencia, Tecnología, Conocimiento e Innovación, 2024). Implementing targeted policy responses such as expanded program budgets, ranked reserve lists for “very good” but unfunded proposals, and structured transitions between Initiation and Regular grants (with gender-sensitive provisions and supports for caregivers) could enhance research continuity and better align national policy with international standards of early-career support.

Respondents' favorable ratings of the ANID application platform and procedural aspects indicate that administrative barriers are not the primary friction in grant-seeking, yet perceived funding probability declines markedly with career stage signaling a perceived funnel where advancement is constrained by limited slots rather than process quality.

### Early-career researchers and gender disparity

As already pointed out, the data reveal a substantial, gendered caregiving burden that likely constrains career progression despite similar overall working hours and reported research time percentages: women are more likely to provide care and spend markedly more hours per week on caregiving, and this differential is associated with a pronounced gap in pursuit of higher-tier competitive funding, as shown by far fewer women applying to FONDECYT Regular grants. These patterns indicate that caregiving imposes opportunity costs that reduce researchers' capacity or willingness to compete for advanced grants, thereby contributing to downstream gender inequities in resources, leadership, and promotion. This individual-level constraint is closely tied to the broader phenomenon of the “child penalty” observed in the Chilean labor market, where becoming a mother leads to a sharp decline in employment, working hours, and labor earnings, with fathers‘ outcomes remaining largely unaffected (Berniell et al., 2021). The need to reconcile work and family responsibilities often pushes Chilean mothers toward a significant increase in labor informality when seeking flexibility, frequently at the expense of long-term career prospects and quality of employment (Eberhard et al., 2023). In the academic sphere, the implicit demand for high dedication and inflexible hours during the critical early-career stage directly limits the capacity of female researchers to meet competitive application criteria, given the primary domestic responsibility for childcare. Furthermore, the organizational structure of Chilean academia itself exacerbates these inequities. Academic organizations have been characterized by a “gendered stratification,” where research is masculinized and established as the privileged practice that determines academic recognition and evaluation (Mandiola et al., 2019). This environment is further complicated by the penetration of managerial logic (managerialism) into universities, which frames academic practices in terms of a hegemonic masculinity, creating systemic barriers that operate independent of individual productivity metrics (Mandiola et al., 2023). Viewed internationally, the unequal impact of parenthood is a known, persistent challenge across STEM fields. Comparative studies confirm that parenthood explains most of the short-term gender productivity gap in academia, primarily because mothers exhibit lower average productivity compared to fathers or non-parents (Morgan et al., 2021). For female scientists, starting a family during a pivotal career stage imposes a significant cost, leading some to leave the profession or to base crucial career decisions on institutional policies concerning paid parental leave and adequate childcare (Powell, 2021). Consequently, the gender gap in high-tier grant applications, such as the one documented here for FONDECYT Regular grants, aligns with a global pattern where structural and cultural factors fundamentally obstruct the advancement of women into elite research careers.

### Research, responsibilities and personal fulfillment: final recommendations

The high levels of satisfaction with intellectual challenge, responsibility, autonomy, and perceived social impact strongly motivate researchers and are likely to contribute to retention. However, these positive intrinsic dimensions coexist with clear dissatisfaction over work–life balance, workload intensity, and compensation, revealing a bifurcated professional experience in which meaningful engagement is undermined by structural stressors. The consistency of this pattern across gender, contract type, and institution suggests system-wide problems rather than isolated pockets of discontent. Therefore, policy responses should prioritize addressing extrinsic deficits through workload reallocation, salary adjustments, clearer career progression pathways, and institutional supports (such as flexible schedules, childcare, and recognition of non-publication contributions) to ensure the motivational strengths of the academic role are not eroded by chronic overload and inadequate remuneration. Specifically, responses should focus on protecting research time for caregivers, offering targeted transition and consolidation funding for applicants with documented care responsibilities, adopting flexible workload and evaluation timelines, and expanding institutional supports such as accessible childcare or caregiving allowances to mitigate the cumulative career penalties observed. This need for institutional reform must be accompanied by greater national investment in research, as Chile's current level of 0.39% of GDP—well-below the OECD average of 2.9%—constrains both scientific development and researchers' working conditions.

### Limitations and projections of the study

Finally, it is crucial to acknowledge the limitations of this study, particularly regarding the sample's representativeness at the national level. Our sample comprises 267 early-career researchers. To contextualize this figure, according to the latest reports from the Chilean Ministry of Science, Technology, Knowledge, and Innovation (Ministerio de Ciencia, Tecnología, Conocimiento e Innovación, 2023), the country has approximately 9,000 active researchers in academia, with a national gender distribution of roughly 35% female and 65% male researchers. While our sample provides valuable exploratory insights, it is not statistically representative of the entire national ecosystem. Notably, our sample exhibits a significant disciplinary bias, with 58.8% of respondents coming from the natural sciences and an overall STEM representation of 71.9%. This skew is largely an unintended consequence of the snowball sampling methodology employed for data collection, which naturally propagated more efficiently through the immediate academic and institutional networks of the research team. Because academic career trajectories, funding opportunities, and precarity profiles in STEM fields can differ significantly from those in the Humanities, Arts, and Social Sciences (HASS; Bozeman and Gaughan, 2011; Ministerio de Ciencia, Tecnología, Conocimiento e Innovación, 2024), our findings may over represent the specific realities of STEM researchers. Future research should aim for stratified sampling to fully capture the disciplinary nuances of the Chilean academic landscape.

## Data Availability

The raw data supporting the conclusions of this article will be made available by the authors, without undue reservation.
